# Supplementing a grain diet with insects instead of fruits sustains the body condition of an omnivorous bird

**DOI:** 10.1002/ece3.10141

**Published:** 2023-05-25

**Authors:** Ojodomo G. Simon, Shiiwua A. Manu, Chima J. Nwaogu, Taiwo C. Omotoriogun

**Affiliations:** ^1^ A. P. Leventis Ornithological Research Institute University of Jos Jos Nigeria; ^2^ Department of Zoology Ahmadu Bello University Zaria Nigeria; ^3^ Department of Zoology University of Jos Jos Nigeria; ^4^ FitzPatrick Institute of African Ornithology University of Cape Town Cape Town South Africa; ^5^ Biotechnology Unit, Department of Biological Sciences Elizade University Ilara‐Mokin Nigeria

**Keywords:** body condition, diet limitation, diet preference, environmental change, environmental seasonality, nutrient limitation, omnivory

## Abstract

Omnivores utilize dietary sources which differ in nutrients, hence dietary limitations due to environmental change or habitat alteration could cause nutrient limitations, and thus deterioration of body condition if omnivory is obligate. We investigated how the body condition of the omnivorous Village weaver *Ploceus cucullatus* (weavers), which forages predominantly on grains, responds to the supplementation of its grain diet with insects instead of fruits. Forty wild‐caught weavers held in aviaries were fed a combination of grains and fruits, or grains and insects ad libitum for 8 weeks. We determined diet preference by recording the number of birds on each diet option per minute for 1 h and the amount of food left‐over after 3 h of foraging. Fortnightly, we assessed indices of body condition including body mass, pectoral muscle, and fat scores, packed cell volume (PCV), and hemoglobin concentration (HBC). We modeled the number of foragers, food left‐over, and body condition indices as functions of diet, while accounting for time (weeks) and sex effects. Grains were the preferred diet, but males ate more fruits and insects than females. Weavers fed on grains and fruits lost body and pectoral muscle mass and accumulated less fat than those fed on grains and insects. This effect was sex‐dependent: females supplemented with fruits lost more pectoral muscle mass than males of the same group and males but not females, supplemented with insects accumulated more fat reserve than those supplemented with fruits. PCV and HBC did not differ between diets but increased over the 8 weeks. Weavers are likely obligate rather than facultative omnivores, with insects as being a more nutritive supplement than fruits. Nutrient limitation arising from environmental change or habitat alteration could impair body condition and affect physiological function to environmental seasonality in obligate omnivores like the weavers.

## Graphical abstract

Diet limitation and the associated nutrient limitation influenced optimal body condition and physiological response to environmental seasonality in an omnivorous song bird.

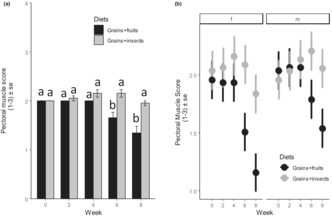

## 1 INTRODUCTION

Omnivory implies nutrient acquisition from plant and animal sources (Agrawal & Klein, 2000; Singer & Bernays, 2003). Dietary sources utilized by omnivores may differ widely in nutrient content, and despite foraging on different food types, many omnivores have a primary or preferred diet. For example, fruit and vegetable diets are protein deprived; hence, most avian omnivores may better be described as granivores, frugivores, or herbivores that incorporate protein‐rich insects into their diets to complement their primary plant diets (Burin et al., 2016; Coll & Guershon, 2002). Others are diet specialists or facultative omnivores, which do not necessarily require supplementation (Griffith et al., 2021). Where omnivory is obligate, deprivation of supplementary diets crucial for body functions will lead to nutrient limitation (Gillespie et al., 2012; Gillespie & McGregor, 2000), whereas facultative omnivores who can derive all the required nutrients from their primary diet may be unaffected by such deprivation (Milne et al., 1996; Milne & Walter, 1997; Trichilo & Leigh, 1988).

Environmental changes capable of altering the availability of specific food types can lead to nutrient limitation with effects on morphology and physiology (Filipiak & Filipiak, 2020; Pierce & McWilliams, 2004; Pryke et al., 2012). For example, dietary protein limitation may lead to the breakdown of protein‐rich tissues including muscles and digestive organs (Krieger et al., 2006; Piersma & Gill, 1998). The ability of animals to temporally adjust body reserves in response to breeding and changes in environmental conditions may also be impaired by diet or nutrient limitation with effects on survival and fitness (Eikenaar et al., 2021; Harrison et al., 2011; Liknes & Swanson, 2011). Moreover, the effects of nutrient limitation may be sex‐specific because of differences in breeding roles (e.g., Mallory et al., 2008), physiology (e.g., Filipiak & Filipiak, 2020), and morphology (e.g., Fernández‐Montraveta & Moya‐Laraño, 2007).

In the absence of diet limitation, animals may still face specific nutrient limitations in nature due to changes in the quality of their preferred diet, the energetic demand of cold conditions, and the occurrence of other energy/nutrient‐demanding periods of the annual cycle such as breeding and molt, resulting in over‐exploitation of body reserves (Andersson et al., 2018; Bravo et al., 2019; Cherel et al., 1994; Murphy & Pearcy, 1993; Swanson, 2010). To maximize nutrient intake during periods of limited nutrient availability, animals may shift between diets or show preference for a given food type (Kwieciński et al., 2017; Lamperti et al., 2014; McWilliams et al., 2004). Such periodic shifts may also be sex‐specific, arising due to differences in morphology (Bravo et al., 2019; Walker et al., 2014), physiology, and breeding roles (Kwieciński et al., 2017; Treidel et al., 2021). In birds, body mass, pectoral muscle, and fat scores indicate physical condition in terms of energy and nutrient reserves (Milenkaya et al., 2013). PCV and HBC, on the contrary, are physiological indices which may indicate part of overall health status in combination with other biochemical indices (Garvin et al., 2007). Omnivorous birds in changing environments are useful surrogates for investigating how diet limitation and/or nutrient limitation due to environmental changes and timing of annual cycle events can influence body condition. In this study, we determined how deprivation of supplementary dietary items affect the body condition (body mass, pectoral muscle score, fat score, PCV, and HBC) of Village weavers. Their omnivorous foraging behaviour makes them amenable to captive conditions and suitable for experimental diet manipulation. This allowed the effects of nutrient limitation (resulting from different levels of dietary supplementation) on body condition to be determined. First, we determined that grains were the preferred diet of Village weavers, and fruits and grains to be the most consumed diets; then, we tested how preference for grains, insects, and fruits may vary over time and between sexes. We expect weavers to show preference for grains compared with fruits and insects during periods of higher energy demands such as under colder environmental conditions. We also expected diet preference to vary between sexes due to their differences in plumage coloration, sex roles, and physiology. Secondly, we compared body condition indices between male and female weavers fed on grains and fruits and those fed on grains and insects over 8 weeks. We expect a deterioration of body condition in weavers on both diet treatments if omnivory is obligate, and a more pronounced deterioration in weavers deprived of the more essential supplementary diet between insects and fruits. We expected differences in how body condition varies over time and between sexes if nutrient demands vary differently between sexes with changes in environmental conditions over time.

## 2 MATERIALS AND METHODS

### 2.1 Study species

The Village weaver *Ploceus cucullatus* is an omnivorous tropical bird found throughout sub‐Saharan Africa (Craig, 2010). In the wild, Village weavers exist in large colonies (varying from a few nests to 100 in one tree) and forage in small groups often around water bodies or human settlements. They thrive well in captivity and are commonly kept as pets (Collias et al., 1986; Fry & Keith, 2004). Village weavers are predominantly granivorous (Collias et al., 1986; Collias & Collias, 1970), but they also forage on insects (Adegoke, 1983; Collias & Collias, 1970) and fruits (Lahti, 2003; Yilangai et al., 2014). The species is polygynous where males build nests but nestlings are predominantly fed by females (Collias & Collias, 1970). Male weavers are larger (average weight = 41.3) and have brighter feathers compared with females (average weight = 33.8), especially during the breeding period (Adegoke, 1983; Borrow & Demey, 2004).

### 2.2 Experimental set‐up

We trapped 20 male and 20 female adult Village weavers using mist nets around the A. P. Leventis Ornithological Research Institute's (APLORI) Amurum Forest Reserve (AFR) (09° 87′ N, 08° 97′ E), Nigeria, between October and November 2020. The birds were trapped shortly after breeding while molting feathers into non‐breeding plumage. Each bird was fitted with a uniquely numbered metal ring and a combination of three color rings. Birds were housed in groups of 10 of equal sexes in four adjacent outdoor aviaries at the APLORI. The aviaries measured 3 × 1 × 2 m with a concrete floor, a metal frame, wire mesh, and a thatched roof made from grass mats. Birds were fed grains, insects, and fruits for 2 weeks before the commencement of the experiment, which lasted a further 8 weeks. Birds were randomly assigned to aviaries and diet treatments were assigned to aviaries systematically, with each set of two adjacent aviaries having alternate diet treatments. Birds in two aviaries were fed crushed grains and fruits, while the other two were fed crushed grains and insects. Two hundred gram (200 g) of each food types were provided in separate trays placed side by side. Food and water were provided ad libitum. All birds were weighed (±0.1 g), scored for pectoral muscle and fat, and sampled for blood to measure PCV and HBC before the diet experiment commenced. Subsequently, birds were sampled fortnightly over 8 weeks. Sampling took place on two consecutive days during each session, with two aviaries of alternate diet treatments sampled per day in a rotating order (Nwaogu et al., 2020).

### 2.3 Diet composition

The grain diet consisted of grass seeds (e.g., Red grass *Themeda triandra*, Rhode grass *Chloris gayana*, Guinea grass *panicum maximum*, Roofing grass *Hyparrhenia involucrata*, and Love grass *Erasgrostis tenella*) available to Village weavers in the wild and crushed cultivated grains (Pearl millet *Pennisetum glaucum*, Sorghum *Sorghum bicolor*, Maize *Zea mays*, and Rice *Oryza sativa*). The macro‐nutrient composition of the grain diet ranged from around 50 to 66% for carbohydrate, 10 to 18% for protein, and 3 to 8% for lipids (Okonwu et al., 2022). The insects' diet consisted of crushed air‐dried grasshoppers and immobilized ants, grasshoppers, and termites caught in the AFR using sweep nets in known foraging patches used by Village weavers with around 2–13% carbohydrate, 43–75% protein, and 6–40% lipids (Oibiokpa et al., 2017). The fruit diet consisted of ripe fruits of Common lantana *Lantana camara,* Sumach plant *Rhus natalensis,* Pawpaw *Carica papaya*, and Watermelon *Citrullus lanatus* and composed of around 6–87% carbohydrate, 0.2–2.6% protein, and 0.1–8% lipids (Chukwuka et al., 2013; Karadaş et al., 2020; Yimer & Tehulie, 2020).

### 2.4 Relative diets preference

We recorded the number of birds feeding on a given food tray at every 60 s interval across the four aviaries within an hour with a telescope stationed 25 m from the aviaries. We carried out observations in the morning (1000–1100 h. GMT) and evening (1600–1700 h. GMT) of three selected days per week. We recorded the identity and sex of foraging individuals throughout the experiment. Also, we weighed (±0.1 g) the amount of food left on each food tray (defined as giving‐up density, hereafter GUD) at 3‐h intervals after food was provided using a digital weighing scale. Hence, we measured GUD at 0900, 1200, 1500, and 1800 h after providing food at 0600 to account for possible shifts in diet preferences during the day.

### 2.5 Diets effects on body mass, pectoral muscle, fat, PCV, and HBC

We sampled all birds randomly per aviary between 0600 and 1000 h, and returned them together after the last bird had been sampled from a given aviary during each sampling session. Birds were held in soft dark cloth bags after capturing from the aviary and after sampling to minimize stress (Nwaogu et al., 2020). We weighed each bird with a digital weighing scale and scored pectoral muscle and fat on a scale of 1–3 and 0–9, respectively (Redfern & Clark, 2001). We also noted whether birds were molting body feathers and flight feathers at the beginning and end of the experiments. We collected 75 μL of blood from each bird into a heparinized micro‐hematocrit tube after puncturing the brachial vein with a needle. The blood sample was then emptied into ethylenediaminetetraacetic acid (EDTA) bottle, stored on ice, and transported to the National Veterinary Research Institute (NVRI) Vom, Plateau State, Nigeria for determination of PCV following methods described by Turkson and Ganyo (2015). To measure the HBC of each bird, micro‐hematocrit tube was filled with 10 μL of whole blood and dropped on the strip of a portable handheld hemoglobinometer and the HBC (g^–1^ dL) was recorded.

### 2.6 Statistical analyses

All analyses were carried out using the statistical software R version 4.0.2 (R Development Core Team 2020). Data were tested for normality and homogeneity of variance using the Shapiro–Wilk normality test and residual plots respectively. Only the number of weavers foraging per diet per minute deviated from a normal distribution. To model relative diet preference, we built a generalized linear model with a Poisson error structure including diet, week, feeding session, sex, and their two‐way interactions as explanatory variables for variation in the number of weavers foraging on each diet per minute. We tested for differences in GUD between diets using a linear model. Diet, week, feeding session (time of day), and their two‐way interaction terms were included in the model as explanatory variables for variation in GUD. To test for the effect of diet on body condition, we accounted for the effects of molt and aviaries on all indices by including molt status and aviary as predictor variables in the model for each body condition index. The explanatory power of both molt and aviaries on variation in all indices was not significant and so were dropped from all our models. Hence, we built linear models for each body condition index with diet, week, sex, and their two‐way interactions as the main explanatory variables. For all models, a post hoc test was used to determine which groups differed significantly from each other. We treated pectoral muscle and fat score as continuous data rather than ordinal data and analyzed them using linear models because their residuals were normally distributed. However, the ordinal logistic regression analysis for pectoral muscle and fat score are similar to linear regression analysis, and are reported as supporting information (Table S6).

## 3 RESULTS

### 3.1 Diet preference

**FIGURE 1:**
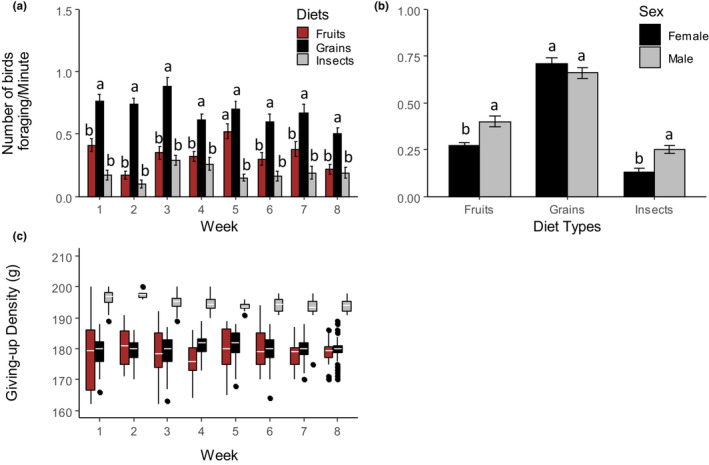
Relative diet preference of Village weavers *Ploceus cucullatus* over 8 weeks of diet treatments. (a) Number of Village weavers foraging per minute (±se) on diet types. (b) Number of males foraging relative to females on diet types. (c) Variation in giving‐up density (GUD) (g) between diet types. Letters above bars indicate a statistically significant difference between groups after post hoc comparisons at *p* < .05. Boxes show group medians. GUD of fruits and grains is significantly (*p* < .05) lower compared with insects across weeks while the GUD of fruits is significantly (*p* < .05) lower compared with grains in Weeks 1 and 4 (Table S2).

Grains were most preferred by weavers throughout the 8 weeks of the experiment followed by fruits and then insects. The number of birds foraging on fruits versus insects differed significantly only at Week 5, while fruits versus grains differed significantly except in Week 5 (Figure 1a, Table 1 & Table S1). More males foraged on fruits and insects than females, but sexes foraged similarly on grains (Figure 1b, Table 1 & Table S1). Insects had the highest GUD throughout the 8 weeks followed by grains and fruits (Figure 1c), but the GUD of fruits was only significantly lower than grains in Weeks 1 and 4 (Figure 1c, Table 1 & Table S2).

**TABLE 1:** Differences in the number of Village weavers *Ploceus cucullatus* foraging on diet types per minute of observation, and giving‐up density (GUD) (g) of diet types over 8 weeks of foraging.

Number of weavers foraging on diet types/minute				GUD (g)				
Variables	df	Chisq	*p*	Variables	df	Sum Sq.	*F*	*p*
Diets	2	542.33	**<.001**	Diets	2	35296.00	614.18	**<.001**
Week	7	54.29	**<.001**	Week	7	338.00	1.68	.110
Sex	1	3.11	.078	Session	1	96.00	3.33	.070
Session	1	223.11	**<.001**	Diets × Week	14	1334.00	3.32	**<.001**
Diets × Week	14	51.75	**<.001**	Diets × Session	2	219.00	3.81	.123
Diets × Sex	2	35.17	**<.001**	Week × Session	7	326.00	1.62	.126
Diets × Session	2	1.86	.394	Residuals	734	21091.00		
Week × Sex	7	29.75	**<.001**					
Week × Session	7	20.36	**.005**					
Sex × Session	1	0.24	.626					

*Note*: Statistically significant effects are highlighted in bold. Session = time of day.

### 3.2 Effects of diet on body condition

**FIGURE 2:**
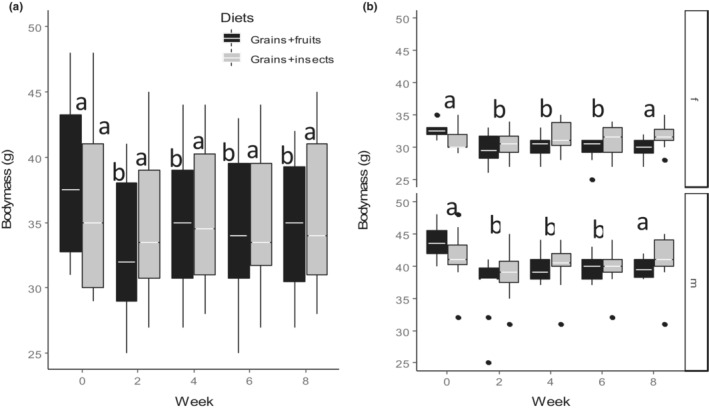
Effect of diet treatment on the body mass (g) of Village weavers *Ploceus cucullatus* over 8 weeks of experiment between (a) Diet types (b) Sexes (f = female, m = male). Boxes show group medians. Boxes for each diet with different alphabets are significantly different while different letters above boxes for sexes indicate a significant difference between weeks after post hoc comparisons at *p* < .05.

The weavers which fed on grains and fruits lost significantly more body mass between Week 0 (before the experiment) and Week 8 (at the end of the experiment) compared with the weavers that were fed on grains and insects (Figure 2a, *F*_4,183_ = 2.63, *p* = .036, Table 2). This pattern was similar between sexes (Figure 2b, *F*_1,183_ = 1.17, *p* = .282, Table 2 & Table S3B). Body mass decreased significantly since the experiment commenced (between Week 0 and Week 2) only in weavers fed on grains and fruits, but weavers fed on grains and insects maintained their body masses throughout the experiment (Figure 2a, Table S3A), and this was similar for males and females (Figure 2b, *F*_4,183_ = 1.29, *p* = .275, Table 2 & Table S4).

**TABLE 2:** Comparison of body mass (g), pectoral muscle score (on a scale of 1–3), and fat score (on a scale of 0–9) between Village weavers *Ploceus cucullatus* fed grains and fruits and grains and insects for 8 weeks of an experiment.

	Body mass (g)				Pectoral muscle score (1–3)			Fat score (0–9)		
Variables	df	Sum Sq.	*F*	*p*	Sum Sq.	*F*	*p*	Sum Sq.	*F*	*p*
Diets	1	10.10	1.33	.250	3.38	37.86	**<.001**	1.81	5.20	**.024**
Weeks	4	229.00	7.55	**<.001**	4.57	12.80	**<.001**	4.60	3.31	**.012**
Sex	1	4241.20	558.90	**<.001**	0.72	8.06	**.005**	3.13	8.99	**.003**
Diets × Weeks	4	79.80	2.63	**.036**	2.97	8.32	**<.001**	1.52	1.09	.361
Diets × Sex	1	8.80	1.17	.282	0.27	3.04	.083	1.48	4.26	**.040**
Weeks × Sex	4	39.20	1.29	.275	0.63	1.76	.138	1.80	1.30	.273
Residuals	183	1388.70			16.34			63.54		

*Note*: Statistically significant effects are highlighted in bold.

**FIGURE 3:**
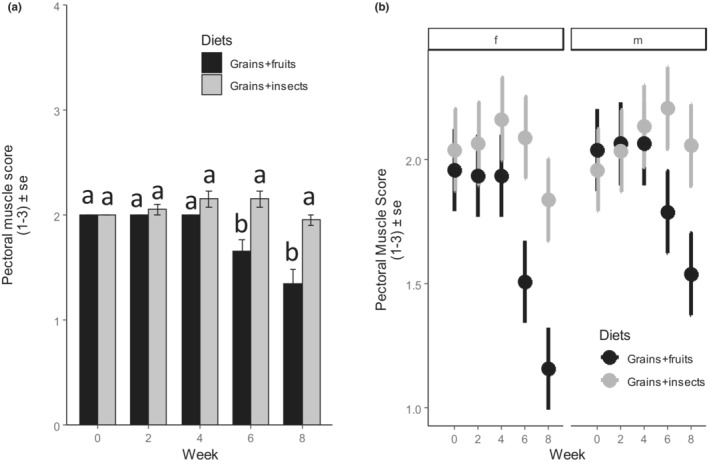
Effect of diet treatment on the pectoral muscle size (on a scale of 1–3) of Village weavers *Ploceus cucullatus* over 8 weeks of experiment between (a) Diet types (b) Sexes (f = female, m = male). Both sexes had higher and similar muscle scores on grains and insects than on grains and fruits diets. Bars with different letters are significantly different after post hoc comparisons at *p* < .05.

Muscle size was similar between the weavers fed on grains and fruit, and those fed on grains and insects before the experiment in Week 0 (Figure 3a), but upon diet restriction, the muscle size of the weavers fed on grains and fruits significantly became lower than those fed on grains and insects (Figure 3a, *F*_4,183_ = 8.32, *p* < .001, Table 2), and this pattern differed marginally between sexes (Figure 3b, *F*_1,183_ = 3.04, *p* = .083, Table 2 & Table S3B): Females fed on grains and fruits lost more pectoral muscle mass than males of the same group. Muscle size decreased significantly during the experiment in Weeks 6 (*t* = 6.88, *p* < .000, Table S3A), and 8 (*t* = 3.70, *p* = .010, Table S3A) in the weavers fed on grains and fruits, but weavers fed on grains and insects maintained their muscle sizes throughout the experiment (Figure 3a, Table S3A). This pattern was similar between males and females (Figure 3B, *F*_4,183_ = 1.76, *p* = .138, Table 2 & Table S4).

**FIGURE 4:**
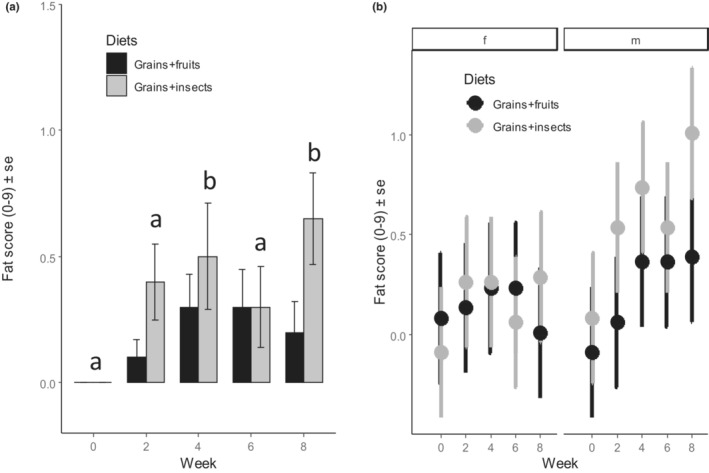
Effect of diet treatment on the fat score (on a scale of 0–9) of Village weavers *Ploceus cucullatus* over 8 weeks of experiment between (a) diet types (b) sexes (f = female, m = male). Males differ in their fat score between diets but females do not. Differences in letter above each pair of bars indicate a significant difference between weeks after post hoc comparisons at *p* < .05.

Overall, the fat score was similar between the weavers fed on grains and fruits, and those fed on grains and insects before the experiment in Week 0 (Figure 4a). During the experiment, the weavers fed on grains and insects significantly accumulated more fat compared with the weavers fed on grains and fruits (Figure 4a), but this was sex‐dependent (Figure 4b, *F*_1,183_ = 4.26, *p* = .040, Table 2). As such, fat scores did not differ significantly (*t* = −0.05, *p* < .001) between diet treatments for females, but males fed on grains and insects accumulated more fat than those fed on grains and fruits (Figure 4b, *t* = −2.94, *p* = .019, Table S3B). For both treatments, fat reserve increased from Weeks 2 to 8 and differed between weeks (Figure 4a, *F*_1,184_ = 3.31, *p* = .012, Table 2 & Table S3A) and this was similar for males and females (*F*_1,183_ = 1.30, *p* = .273, Table 2 & Table S4).

**FIGURE 5:**
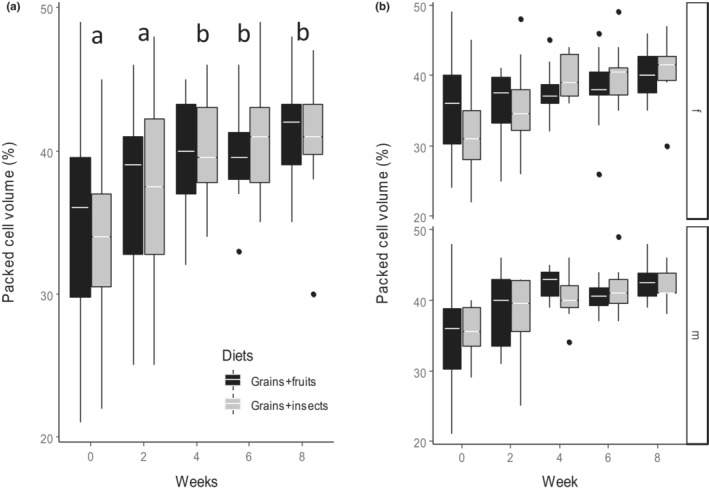
Effect of diet treatment on the packed cell volume (PCV) (%) of Village weavers *Ploceus cucullatus* over 8 weeks of experiment between (a) diet types (b) sexes (f = female, m = male). Boxes show group medians. PCV did not differ between diets but differed between weeks for both diets and sexes. Males had higher PCV than females throughout the experiment on both diets. Differences in letter above each pair of boxes indicate a significant difference between weeks after post hoc comparisons at *p* < .05.

Diet treatment had no effect on PCV (*F*_1,183_ = 0.06, *p* = .808, Table 3); however, PCV increased significantly over the course of the experiment in both diet treatments (Figure 5a, *F*_4,183_ = 12.54, *p* < .001, Table 3 & Table S5) and this was similar between males and females (Figure 5b, *F*_4,183_ = 0.09, *p* = .986, Table 3).

**TABLE 3:** Comparison of packed cell volume (PCV) (%) and hemoglobin concentration (HBC) (g^−1^ dL) between Village weavers *Ploceus cucullatus* fed grains and fruits and grains and insects for 8 weeks of an experiment.

	PCV (%)				HBC (g^−1^ dL)		
Variables	df	Sum Sq.	*F*	*p*	Sum Sq.	*F*	*p*
Diets	1	1.40	0.06	.808	0.73	0.28	.597
Weeks	4	1224.40	12.54	**<.001**	170.37	16.34	**<.001**
Sex	1	239.80	9.82	**.002**	31.92	12.25	**<.001**
Diets × Weeks	4	48.90	0.50	.735	2.77	0.27	.899
Diets × Sex	1	8.60	0.35	.554	0.83	0.32	.573
Weeks × Sex	4	8.50	0.09	.986	0.96	0.09	.985
Residuals	183	4467.30			477		

*Note*: Statistically significant effects are highlighted in bold.

**FIGURE 6:**
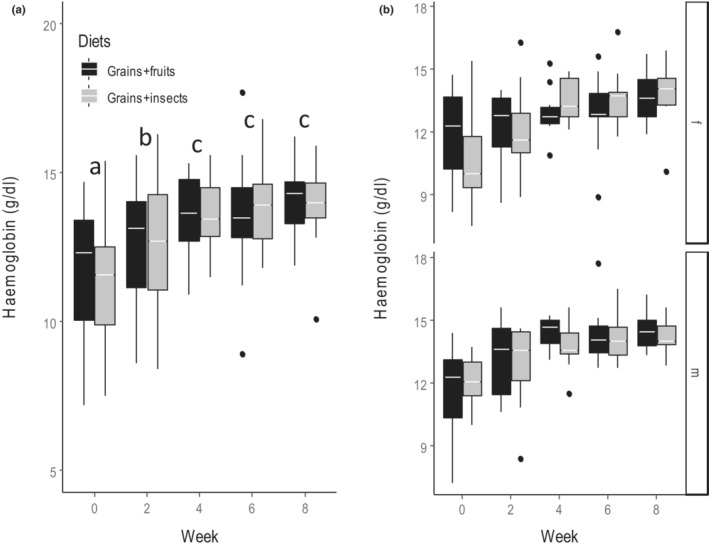
Effect of diet treatment on the hemoglobin concentration (HBC) (g^−1^ dL) of Village weavers *Ploceus cucullatus* over 8 weeks of experiment between (a) diet types and (b) sexes (f = female, m = male). Boxes show group medians. HBC did not differ between diet treatment but differed between weeks for both diets and sexes. Males had higher *HBC* than females throughout the experiment on both diets. Differences in letter above each pair of boxes indicate a significant difference between weeks after post hoc comparisons at *p* < .05.

Similarly, diet had no effect on HBC (*F*_1,183_ = 0.28, *p* = .597, Table 2), but HBC increased significantly during the experiment in both diet treatments (Figure 6a, *F*_4,183_ = 16.34, *p* < .001, Table 3 & Table S5). This pattern was similar between sexes (Figure 6b, *F*_4,183_ = 0.09, *p* = .985, Table 3).

## 4 DISCUSSION

We tested how the body condition of wild‐caught captive Village weavers responded to supplementation of their predominantly grain diet with either fruits or insects. Village weavers prefer grains compared with fruits and insects as indicated by observed foraging frequencies on the diet types per unit time, and GUD. Weavers fed with a diet of grains supplemented with insects lost less body mass, maintained pectoral muscle, and accumulated higher fat reserves over the 8 weeks of the experiment compared with those whose grain diet was supplemented with fruits. PCV and HBC did not differ between diet treatments but increased over time in both diet treatments.

The observed preference for grains is consistent with existing knowledge that Village weavers are largely granivores despite being described as omnivores (Adegoke, 1983; Collias & Collias, 1970). Grains and seeds are richer in carbohydrates and fatty acids (which are highly concentrated energy sources) than fruits and insects (Dimiceli et al., 2007; Karasov & Martinez del Rio, 2007). Therefore, the consistently higher number of Village weavers foraging on grains relative to fruits and insects suggests that a diet of grains is more crucial for maximizing their daily energy requirements relative to fruits and insects. However, despite this preference for grains, weavers foraged on fruits and insects probably to obtain specific vitamins and minerals or nutrients such as carotenoids (a source of yellow, red, or orange pigments), and proteins which are less available or unavailable in grains (Bairlein, 2002; Linville & Breitwisch, 1997; Walker et al., 2014). Thus, a grain diet alone might not be optimal or contain sufficient quantities of required nutrients and has to be complemented with fruits and insects by weavers and other granivores.

Relative diet preference may simply indicate the amount of each food item intake, or effort required to meet daily nutrient/energy requirements. For example, based on the absolute mass of food items consumed from GUD estimates, the weavers consumed as much fruits as grains weekly, suggesting that they were much more efficient in handling the fruits compared with grains. Note, however, that the observed similarity in GUD between grains and fruits might be partly influenced by evaporative water loss from the fruits during exposure in the aviaries, so overall, weavers may have consumed more grains than fruits. The lower GUD of insects on the other hand, may simply imply that the weavers only need to feed on a small quantity of insects to meet their daily dietary protein requirements (Karasov & Levey, 1990; Klasing, 1998). Our observation that males fed more on fruits (and insects) than females is consistent with the idea that colorful males target carotenoid‐rich foods in the wild (Walker et al., 2014). We presume this is because of the differential nutrient preference for carotenoids and protein to be incorporated into molting feathers since males are more brightly colored than females and were actively molting at the time of the experiment (Murphy & Pearcy, 1993). The observed preference for fruits and insects by the males in our study is unlikely due to dominance of males over females because both sexes fed equally on grains supplied ad libitum.

Body mass was found to decrease for weavers on both diet treatments. This implies that captivity and/or the diet combination provided in this study are suboptimal for the Village weavers. Body mass fluctuations could also be driven by natural events like circannual cycles. It is however worthy of note that the decrease in body mass was more pronounced in the fruit‐supplemented weavers, suggesting that diet supplemented by insect may better satisfy nutrient requirements compared with diets supplemented by fruit. Fat and muscles including the digestive organs and pectoral muscles account for around 50% and 25% of the total body mass of birds, respectively (Labocha & Hayes, 2012; Ndlovu et al., 2017). Our results show that body mass loss in the weavers fed with grains and fruits is unlikely due to fat loss, but due to pectoral muscle breakdown. The weavers increased fat reserves over time during the experiment probably because of the decreasing temperature in our study area from November to February annually. This period is characterized by a cold–dry spell with temperatures as low as 3°C in the nights and early mornings when birds need to endure temporary bouts of starvation and yet maintain their body temperature. Protein‐rich pectoral muscles are part of the non‐fat component of body mass, which can be broken down to provide amino acids needed for body maintenance, a plausible explanation for the weavers not to lose fat but significantly deplete muscle tissues. Several studies have reported the loss of body mass due to a reduction in the size of digestive organs rather than pectoral muscles in long‐distance migratory birds because muscles are needed for long‐distance endurance flight (Krieger et al., 2006; Lindstrom et al., 2000; McWilliams & Karasov, 2005; Pierce & McWilliams, 2004; Piersma et al., 1999). On the contrary, weavers in captivity do not need to fly long distances but they needed to continue foraging on a potential suboptimal food source, thereby depleting pectoral muscles rather than reducing their digestive tract. Protein limitation in the fruit‐fed weavers may be exacerbated by molting requirements, but this may not be the case in this study because molt status showed no effect on the observed loss of pectoral muscle mass. Around 90% of bird feathers are made up of keratin which is composed of amino acids derived from protein (Stettenheim, 2000). During molts, around 25% of protein mass is depleted and the demand for dietary protein increases (McWilliams, 2008). Having less protein available, the weavers fed on grains and fruits diet may have catabolized their protein reserve to meet their molt protein requirements. Moreover, unknown to us, the birds may have arrested molt due to protein deprivation (Gosler, 1991) since we took note of molt status only at the beginning and end of the experiment. Females deprived of insects lost more muscle mass than males on the same treatment. It is likely that the larger body size of males influenced the rate at which muscle tissues are depleted since they could have higher protein reserves than females (Piersma, 1984). Furthermore, female weavers are responsible for provisioning nestlings; therefore, the need to compensate for increased nutrient demands incurred by laying eggs, and nourishing nestlings and fledglings with protein‐rich diets may increase the protein requirement of the females (Collias & Collias, 1970; Houston, 1998) and thus could result in muscle depletion.

Variation in body fat may be due to environmental conditions over time rather than diet treatment, but diet may have influenced the intensity of fat accumulation in response to environmental change. We found increased fat reserves in the weavers fed on both diet treatments, but the male weavers fed on grains and insects increased fat reserves more than those fed on grains and fruits. An increase in fat reserves in response to cold temperature has been reported in several species of temperate birds (Brodin et al., 2017; Goławski et al., 2015). Energy demands for heat production increase with decreasing temperatures (Goławski et al., 2015). Therefore, birds must accumulate fat for both energy requirement and insulation during periods of low temperature. Similar increases in body fat and mass were observed in Common Bulbuls *Pycnonotus barbatus* held in captivity in the same study area over the same time of year (Nwaogu, 2019). Nonetheless, it is unclear why males fed on grains and insects gained more fat than those fed with grains and fruits because simple sugars from fruits are expected to allow de novo production and accumulation of fat as reported for migrants, for example, White‐throated Sparrows *Zonotrichia albicollis* (Klasing, 1998; Smith & McWilliams, 2009).

The observation that diet did not affect PCV, but that PCV increased over time regardless of diet treatments suggests that factors other than diet such as humidity, photoperiod, and temperature may be driving variation in PCV (Fair et al., 2007). An increase in PCV can occur when blood plasma is used to disperse body heat in response to cold temperature, thus making the blood more concentrated (Dawson & Bortolotti, 1997a, 1997b). This is consistent with the cold temperatures during the study period. Similarly, Swanson (1990) and Abelenda et al. (1993) found PCV to increase as temperature decreases due to the need to increase heat production in cold temperatures (Carey & Morton, 1976). The lack of a diet effect on PCV was unexpected; however, it is consistent with other studies that found no diet‐related differences in PCV in American Kestrel *Falco sparverius* (Dawson & Bortolotti, 1997a, 1997b) and House Sparrows *Passer domesticus* (Gavett & Wakeley, 1986). The higher PCV in males than females may result from sex hormones. Specifically, androgens, which support sperm production, are known to increase the production of red blood cells in male birds (Shahani et al., 2009). Variation in PCV and HBC followed the same pattern. This is not surprising because HBC correlates positively with PCV in Passerines (Velguth et al., 2010). Thus, an increase in PCV in response to cold temperature and due to androgen effect in males might have resulted in a corresponding pattern in HBC.

This study demonstrates that environmental changes capable of altering the availability of different food types (especially insects) can affect the maintenance of body condition and other physiological processes. Obligate omnivores such as the Village weaver, which depends on several food sources to meet its daily nutrient requirement, are particularly vulnerable. Moreover, omnivores may be more vulnerable to environmental change despite their diverse foraging options due to their complex diet combination (Burin et al., 2016).

## AUTHOR CONTRIBUTIONS

**Ojodomo Godday Simon:** Conceptualization (lead); data curation (lead); formal analysis (lead); funding acquisition (lead); investigation (lead); methodology (equal); software (lead); validation (equal); visualization (lead); writing – original draft (lead); writing – review and editing (lead). **Shiiwua A. Manu:** Conceptualization (lead); supervision (supporting); validation (equal); writing – original draft (equal); writing – review and editing (equal). **Chima Nwaogu:** Conceptualization (lead); data curation (lead); formal analysis (lead); investigation (equal); methodology (lead); software (lead); supervision (supporting); validation (equal); visualization (lead); writing – original draft (equal); writing – review and editing (equal). **Taiwo Crossby Omotoriogun:** Conceptualization (lead); data curation (equal); formal analysis (equal); methodology (equal); software (equal); supervision (lead); validation (equal); visualization (equal); writing – original draft (equal); writing – review and editing (equal).

## CONFLICT OF INTEREST STATEMENT

The authors declare no conflict of interest.

## Supporting information


Appendix S1.

## Data Availability

Data are deposited in Dryad Digital Repository: https://doi.org/10.5061/dryad.4b8gththx.
