# Supplementary material for: Supplementing a grain diet with insects instead of fruits sustains the body condition of an omnivorous bird
Source: Ecol Evol. 2023 May 25;13(5):e10141. doi: 10.1002/ece3.10141 (PMC10213486; doi:10.1002/ece3.10141)
Supplement: Supplementary file 1 — Appendix S1. [file ECE3-13-e10141-s001.docx]

**Supporting Information**

**SUPPLEMENTING A GRAIN DIET WITH INSECTS INSTEAD OF FRUITS SUSTAIN THE BODY CONDITION OF AN OMNIVOROUS BIRD**

Ojodomo G. Simon ^1, 2*^, Shiiwua A. Manu ^1, 3^, Chima J. Nwaogu ^1, 4^, Taiwo C. Omotoriogun ^1, 5^

^1^A. P. Leventis Ornithological Research Institute, University of Jos, P.O. Box 13404, Nigeria

^2^Department of Zoology, Ahmadu Bello University, Zaria, Nigeria

^3^Department of Zoology, University of Jos, Jos, Nigeria

^4^FitzPatrick Institute of African Ornithology, University of Cape Town, South Africa

^5^Biotechnology Unit, Department of Biological Sciences, Elizade University, P.M.B. 002, Ilara-Mokin, Nigeria

*Correspondence: simongodday34@gmail.com

**ORCID: Ojodomo Godday Simon** [0000-0003-1235-1804](https://orcid.org/0000-0002-4623-2355)

**ORCID:** Chima Josiah **Nwaogu** [0000-0002-4623-2355](https://orcid.org/0000-0002-4623-2355)

**ORCID:** Taiwo Crossby Omotoriogun 0000-0001-5678-2687

Table S1: Post hoc summary of pairwise differences (Table 1) in number of Village weavers *Ploceus cucullatus* foraging per diet, per minute across 8 weeks.

| **Variables** | **Pairwise difference** | **Estimate** | **Error** | ***Z*** | ***p*** |
| --- | --- | --- | --- | --- | --- |
| Diets*Week | Fruits 1 - Grains 1 | -0.57 | 0.13 | -4.18 | **.006** |
|  | Fruits 1 - Insects 1 | 0.94 | 0.22 | 4.16 | **.007** |
|  | Grains 1 - Insects 1 | 1.51 | 0.20 | 7.51 | **< .001** |
|  | Fruits 2 - Grains 2 | -1.42 | 0.19 | -7.19 | **< .001** |
|  | Fruits 2 - Insects 2 | 0.58 | 0.30 | 1.91 | .966 |
|  | Grains 2- Insects 2 | 2.01 | 0.25 | 7.92 | **< .001** |
|  | Fruits 3 - Grains 3 | -0.91 | 0.14 | -6.35 | **< .001** |
|  | Fruits 3 - Insects 3 | 0.26 | 0.19 | 1.34 | .999 |
|  | Grains 3 - Insects 3 | 1.17 | 0.15 | 7.40 | **< .001** |
|  | Fruits 4 - Grains 4 | -0.67 | 0.15 | -4.29 | **.004** |
|  | Fruits 4 - Insects 4 | 0.28 | 0.20 | 1.38 | .999 |
|  | Grains 4- Insects 4 | 0.96 | 0.17 | 5.54 | **< .001** |
|  | Fruits 5 - Grains 5 | -0.30 | 0.13 | -2.34 | .789 |
|  | Fruits 5 - Insects 5 | 1.29 | 0.23 | 5.56 | **< .001** |
|  | Grains 5- Insects 5 | 1.59 | 0.21 | 7.44 | **< .001** |
|  | Fruits 6 - Grains 6 | -0.66 | 0.16 | -4.03 | **.012** |
|  | Fruits 6 - Insects 6 | 0.79 | 0.25 | 3.07 | .249 |
|  | Grains 6 - Insects 6 | 1.45 | 0.22 | 6.39 | **< .001** |
|  | Fruits 7 - Grains 7 | -0.62 | 0.16 | -3.88 | **.021** |
|  | Fruits 7 - Insects 7 | 0.62 | 0.27 | 2.32 | .802 |
|  | Grains 7 - Insects 7 | 1.25 | 0.24 | 5.10 | **.001** |
|  | Fruits 8 - Grains 8 | -0.85 | 0.19 | -4.46 | **.002** |
|  | Fruits 8 - Insects 8 | 0.20 | 0.25 | 0.78 | 1.000 |
|  | Grains 8 - Insects 8 | 1.05 | 0.20 | 5.05 | **< .001** |
| Diets*Sex | Fruits Female - Fruits Male | -0.37 | 0.10 | -3.77 | **.002** |
|  | Grains Female - Grains Male | 0.04 | 0.05 | 0.92 | .941 |
|  | Insects Female - Insects Male | -0.60 | 0.14 | -4.34 | **< .002** |

Statistically significant effects are highlighted in bold. Session = time of day.

Table S2: Post hoc summary of pairwise difference (Table 1) in Giving-up density (g) between diet types across 8 weeks of foraging by Village weavers *Ploceus cucullatus*.

| **Variables** | **Pairwise difference** | **Estimate** | **Error** | ***df*** | ***t*** | ***p*** |
| --- | --- | --- | --- | --- | --- | --- |
| Diets*Weeks | Fruits 1 - Grains 1 | -6.14 | 1.34 | 734 | -4.58 | **.001** |
|  | Fruits 1 - Insects 1 | -23.45 | 1.55 | 734 | -15.16 | **< .001** |
|  | Grains 1 - Insects 1 | -17.31 | 1.34 | 734 | -12.91 | **<. 001** |
|  | Fruits 2 - Grains 2 | 1.58 | 1.34 | 734 | 1.18 | 1 |
|  | Fruits 2 - Insects 2 | -16.62 | 1.55 | 734 | -10.74 | **< .001** |
|  | Grains 2 - Insects 2 | -18.20 | 1.34 | 734 | -13.58 | **< .001** |
|  | Fruits 3 - Grains 3 | 0.27 | 1.34 | 734 | 0.20 | 1 |
|  | Fruits 3 - Insects 3 | -15.54 | 1.55 | 734 | -10.04 | **< .001** |
|  | Grains 3 - Insects 3 | -15.81 | 1.34 | 734 | -11.79 | **< .001** |
|  | Fruits 4 - Grains 4 | -5.22 | 1.34 | 734 | -3.90 | **.021** |
|  | Fruits 4 - Insects 4 | -18.25 | 1.55 | 734 | -11.79 | **< .001** |
|  | Grains 4 - Insects 4 | -13.02 | 1.34 | 734 | -9.71 | **< .001** |
|  | Fruits 5 - Grains 5 | -0.81 | 1.34 | 734 | -0.60 | 1 |
|  | Fruits 5 - Insects 5 | -13.58 | 1.55 | 734 | -8.77 | **< .001** |
|  | Grains 5 - Insects 5 | -12.77 | 1.34 | 734 | -9.53 | **< .001** |
|  | Fruits 6 - Grains 6 | 0.00 | 1.34 | 734 | 0.00 | 1.000 |
|  | Fruits 6 - Insects 6 | -14.95 | 1.55 | 734 | -9.66 | **< .001** |
|  | Grains 6 - Insects 6 | -14.95 | 1.34 | 734 | -11.16 | **< .001** |
|  | Fruits 7 - Grains 7 | -1.10 | 1.34 | 734 | -0.82 | 1 |
|  | Fruits 7 - Insects 7 | -14.95 | 1.55 | 734 | -9.66 | **< .001** |
|  | Grains 7 - Insects 7 | -13.85 | 1.34 | 734 | -10.33 | **< .001** |
|  | Fruits 8 - Grains 8 | -0.87 | 1.34 | 734 | -0.65 | 1 |
|  | Fruits 8 - Insects 8 | -15.54 | 1.55 | 734 | -10.04 | **< .001** |
|  | Grains 8 - Insects 8 | -14.66 | 1.34 | 734 | -10.94 | **< .001** |

Statistically significant effects are highlighted in bold.

Table S3: Post hoc summary of pairwise differences (Table 2) in body mass (g), pectoral muscle score (on a scale of 1-3), and fat score (on a scale of 0-9) between grains and fruits and grains and insects fed Village weavers *Ploceus cucullatus* across weeks and between weeks within grains and fruit and grains and insect fed weavers.

| **Pairwise**  **difference** | | | **Body mass (g)** | | | | **Table S3A**  **Pectoral muscle score (1-3)** | | | | **Fat score (0-9)** | | | | | |
| --- | --- | --- | --- | --- | --- | --- | --- | --- | --- | --- | --- | --- | --- | --- | --- | --- |
|  |  | |  | | | |  | | | | **pairwise** |  |  |  |  |  |
| **Week** | **Diet** | **Diet** | **Estimate** | **Error** | ***t*** | ***p*** | **Estimate** | **Error** | ***t*** | ***p*** | ***of weeks*** | **Estimate** | **Error** | ***t*** | ***p*** |  |
| 0 | GF | GI | 1.66 | 0.89 | 1.87 | .688 | 0.02 | 0.09 | 0.17 | 1.000 | 0 – 2 | -0.25 | 0.13 | -1.89 | .324 |  |
| 2 | GF | GI | - 1.50 | 0.89 | -7.43 | .769 | -0.03 | 0.09 | -0.34 | 1.000 | 0 – 4 | -0.4 | 0.13 | -3.03 | **.023** |  |
| 4 | GF | GI | - 1.19 | 0.87 | -1.35 | .941 | -0.13 | 0.09 | -0.38 | 0.930 | 0 – 6 | -0.3 | 0.13 | -2.27 | .157 |  |
| 6 | GF | GI | - 0.99 | 0.89 | -1.12 | .982 | -0.48 | 0.09 | -5.03 | **< .001** | 0 – 8 | -0.42 | 0.13 | -3.22 | **.013** |  |
| 8 | GF | GI | -1.55 | 0.88 | -1.76 | .759 | -0.58 | 0.09 | -6.07 | **< .001** | 2 – 4 | -0.15 | 0.13 | -1.13 | .786 |  |
| **Diet** | **Week** | **Week** |  |  |  |  |  |  |  |  | 2 – 6 | -0.05 | 0.13 | -0.37 | .996 |  |
| GF | 0 | 2 | 4.90 | 0.87 | 5.62 | **<.000** | 0.00 | 0.09 | 0.00 | 1.000 | 2 – 8 | -0.17 | 0.13 | -1.32 | .674 |  |
|  | 0 | 4 | 3.40 | 0.87 | 3.90 | **.005** | 0.00 | 0.09 | 0.00 | 1.000 | 4 – 6 | 0.10 | 0.13 | 0.75 | .942 |  |
|  | 0 | 6 | 3.60 | 0.87 | 4.13 | **.002** | 0.35 | 0.09 | 3.70 | **.010** | 4 – 8 | -0.02 | 0.13 | -0.19 | .999 |  |
|  | 0 | 8 | 3.55 | 0.87 | 4.07 | **.003** | 0.65 | 0.09 | 6.87 | **< .000** | 6 – 8 | -0.12 | 0.13 | -0.94 | .877 |  |
| GI | 0 | 2 | 1.70 | 0.88 | 1.93 | .647 | -0.05 | 0.09 | -0.52 | 1.000 |  |  |  |  |  |  |
|  | 0 | 4 | 0.55 | 0.88 | 0.62 | .999 | -0.15 | 0.09 | -1.58 | .853 |  |  |  |  |  |  |
|  | 0 | 6 | 0.95 | 0.88 | 1.07 | .986 | -0.15 | 0.09 | -1.58 | .853 |  |  |  |  |  |  |
|  | 0 | 8 | 1.89 | 0.89 | 2.13 | .503 | 0.05 | 0.09 | 0.52 | 1.000 |  |  |  |  |  |  |
|  |  |  |  |  |  |  |  |  |  |  |  |  |  |  |  |  |
| **Table S3B**  **Body mass (g) Pectoral muscle score (1-3) Fat score (0-9)** | | | | | | | | | | | | | | | | |
| **Variable** | **Pairwise difference** | | **Estimate** | **error** | ***t*** | ***p*** | **Estimate** | **Error** | ***t*** | ***p*** | **Estimate** | **Error** | ***t*** | ***p*** |  |  |
| Diet*Sex | G+F. f - G+I. f | | -0.45 | 0.39 | -1.14 | .665 | -0.32 | 0.06 | -4.96 | **<.001** | -0.01 | 1.83 | -0.04 | 1.000 |  |  |
|  | G+F.f - G+F. m | | -9.21 | 0.39 | -23.35 | **<.001** | -0.22 | 0.06 | -3.48 | **.003** | -0.09 | 1.83 | -0.74 | .877 |  |  |
|  | G+F. f - G+I. m | | -9.66 | 0.55 | -17.32 | **<.001** | -0.39 | 0.06 | -6.43 | **<.001** | -0.44 | 1.83 | -3.72 | **.002** |  |  |
|  | G+I. f - G+F. m | | -8.76 | 0.55 | -15.70 | **<.001** | 0.09 | 0.07 | 1.25 | .590 | -0.08 | 1.83 | -0.60 | .932 |  |  |
|  | G+I. f - G+I. m | | -9.21 | 0.39 | -23.35 | **<.001** | -0.07 | 0.06 | -1.08 | .701 | -0.44 | 1.83 | -3.25 | **.007** |  |  |
|  | G+F. m -G+I.m | | -O.45 | 0.39 | -1.14 | .665 | -0.17 | 0.06 | -0.70 | **.031** | -0.35 | 1.83 | -2.94 | **.019** |  |  |

Statistically significant effects are highlighted in bold.

G+F = grains and fruits, G+I = grains and insects, f = female. M = male.

Table S4: Post hoc summary of pairwise differences (Table 2) in body mass (g), pectoral muscle score (on a scale of 1-3), and fat score (on a scale of 0-9) of the interaction term between sex and weeks

| **Pairwise difference Body mass (g) Pectoral muscle score (1-3) Fat score (0-9)** | | | | | | | | | | | | |
| --- | --- | --- | --- | --- | --- | --- | --- | --- | --- | --- | --- | --- |
| **Weeks*Sex** | **Estimate** | **Error** | ***t*** | ***p*** | **Estimate** | **Error** | ***T*** | ***p*** | **Estimate** | **Error** | ***t*** | ***p*** |
| f 0 - f 2 | 3.30 | 0.62 | 5.29 | **<.001** | -0.02 | 0.06 | -0.37 | 1.000 | -0.25 | 0.13 | -1.89 | .673 |
| f 0 - f 4 | 1.98 | 0.62 | 3.16 | .**055** | -0.07 | 0.06 | -1.11 | .982 | -0.40 | 0.13 | -3.03 | .080 |
| f 0 - f 6 | 2.27 | 0.62 | 3.64 | **.012** | 0.10 | 0.06 | 1.48 | .896 | -0.30 | 0.13 | -2.27 | .411 |
| f 0 - f 8 | 1.77 | 0.62 | 2.84 | .128 | 0.35 | 0.06 | 5.19 | **<.001** | -0.42 | 0.13 | -3.22 | **.047** |
| m 0 - m 2 | 3.30 | 0.62 | 5.29 | **<.001** | -0.02 | 0.06 | -0.37 | 1.000 | -0.25 | 0.13 | -1.89 | .673 |
| m 0 - m 4 | 1.98 | 0.62 | 3.16 | **.055** | -0.07 | 0.06 | -1.11 | .982 | -0.40 | 0.13 | -3.03 | .080 |
| m 0 - m 6 | 2.27 | 0.62 | 3.64 | **.012** | 0.10 | 0.06 | 1.48 | .896 | -0.30 | 0.13 | -2.27 | .411 |
| m 0 - m 8 | 1.77 | 0.62 | 2.84 | .128 | 0.35 | 0.06 | 5.19 | **<.001** | -0.42 | 0.13 | -3.22 | **.047** |
| f 2 - f 4 | -1.32 | 0.62 | -2.12 | .512 | -0.05 | 0.06 | -0.74 | .999 | -0.15 | 0.13 | -1.13 | .980 |
| f 2 - f 6 | -1.02 | 0.62 | -1.64 | .824 | 0.12 | 0.06 | 1.85 | .698 | -0.05 | 0.13 | -0.37 | 1.000 |
| f 2 - f 8 | -1.52 | 0.62 | -2.44 | .305 | 0.37 | 0.06 | 5.56 | **<.001** | -0.17 | 0.13 | -1.32 | .946 |
| m 2 - m 4 | -1.32 | 0.62 | -2.12 | .512 | -0.05 | 0.06 | -0.74 | .999 | -0.15 | 0.13 | -1.13 | .980 |
| m 2 - m 6 | -1.02 | 0.62 | -1.64 | .824 | 0.12 | 0.06 | 1.85 | .698 | -0.05 | 0.13 | -0.37 | 1.000 |
| m 2 - m 8 | -1.52 | 0.62 | -2.44 | .305 | 0.37 | 0.06 | 5.56 | **<.001** | -0.17 | 0.13 | -1.32 | .946 |
| f 4 - f 6 | 0.30 | 0.62 | 0.48 | 1.000 | 0.17 | 0.06 | 2.59 | .226 | 0.10 | 0.13 | 0.75 | .999 |
| f 4 - f 8 | -0.20 | 0.62 | -0.32 | 1.000 | 0.42 | 0.06 | 6.30 | **<.001** | -0.02 | 0.13 | -0.18 | 1.000 |
| m 4 - m 6 | 0.30 | 0.62 | 0.48 | 1.000 | 0.17 | 0.06 | 2.59 | .226 | 0.10 | 0.13 | 0.75 | .999 |
| m 4 - m 8 | -0.20 | 0.62 | -0.32 | 1.000 | 0.42 | 0.06 | 6.30 | **<.001** | -0.02 | 0.13 | -0.18 | 1.000 |
| f 6 - f 8 | -0.50 | 0.62 | -0.80 | .998 | 0.25 | 0.06 | 3.71 | **.010** | -0.12 | 0.13 | -0.94 | .994 |
| m 6 - m 8 | -0.50 | 0.62 | -0.80 | .998 | 0.25 | 0.06 | 3.71 | **.010** | -0.12 | 0.13 | -0.94 | .994 |

Statistically significant effects are highlighted in bold.

Table S5: Post hoc summary of pairwise differences (Table 3) in packed cell volume (PCV) (%) and (haemoglobin concentration (HBC) (g ^-1^dl) between weeks in Village weavers *Ploceus cucullatus* fed grains and fruits and grains and insects for 8 weeks.

| **Pairwise Difference** | | **PCV (%)** | | | | **HBC (g ^-1^dl)** | | | |
| --- | --- | --- | --- | --- | --- | --- | --- | --- | --- |
| **Week** | **Week** | **estimate** | **Error** | ***t*** | ***P*** | **estimate** | **Error** | ***t*** | ***p*** |
| **0** | **2** | -2.42 | 1.10 | -2.19 | .186 | -1.04 | 0.36 | -2.88 | **.036** |
| 0 | 4 | -5.50 | 1.10 | -4.97 | **<.000** | -2.10 | 0.36 | -5.82 | **<.001** |
| 0 | 6 | -5.52 | 1.10 | -5.00 | **<.000** | -2.20 | 0.36 | -6.11 | **<.001** |
| 0 | 8 | -6.75 | 1.10 | -6.11 | **<.000** | -2.47 | 0.36 | -6.86 | **<.001** |
| 2 | 4 | -3.07 | 1.10 | -2.78 | **.046** | -1.06 | 0.36 | -2.94 | **.030** |
| 2 | 6 | -3.10 | 1.10 | -2.80 | **.044** | -1.16 | 0.36 | -3.23 | **.013** |
| 2 | 8 | -4.32 | 1.10 | -3.91 | **.001** | -1.43 | 0.36 | -3.98 | **<.001** |
| 4 | 6 | -0.02 | 1.10 | -0.02 | **.000** | -0.10 | 0.36 | -0.29 | .998 |
| 4 | 8 | -1.25 | 1.10 | -1.13 | .790 | -0.37 | 0.36 | -1.03 | .837 |
| 6 | 8 | -1.22 | 1.10 | -1.10 | .802 | -0.27 | 0.36 | -0.74 | .945 |

Statistically significant effects are highlighted in bold.

Table S6: Analysis of deviance output of ordinal logistic regression model of pectoral muscle score (on a scale of 1-3) and fat score (on a scale of 0-9) of Village weavers *Ploceus cucullatus* fed grains and fruits and grains and insects for 8 weeks.

|  | **Pectoral muscle score (1-3)** | | | **Fat score (0-9)** | | |
| --- | --- | --- | --- | --- | --- | --- |
| **Variables** | ***df*** | ***Chisq*** | ***P*** | ***df*** | ***Chisq*** | ***p*** |
| Diets | 1 | 37.18 | **< .001** | 1 | 3.82 | .050 |
| Weeks | 4 | 40.23 | **< .001** | 4 | 22.10 | **< .001** |
| Sex | 1 | 7.68 | **.005** | 1 | 8.84 | **.003** |
| Diets*Weeks | 4 | 20.29 | **< .001** | 4 | 4.04 | .400 |
| Diets*Sex | 1 | 2.29 | .130 | 1 | 3.52 | .061 |
| Weeks*Sex | 4 | 1.31 | .859 | 4 | 3.60 | .463 |

Statistically significant effects are highlighted in bold.
